# Activation of antioxidative and detoxificative systems in *Brassica juncea* L. plants against the toxicity of heavy metals

**DOI:** 10.1038/s41598-021-01827-w

**Published:** 2021-11-16

**Authors:** Arleta Małecka, Agnieszka Konkolewska, Anetta Hanć, Liliana Ciszewska, Aleksandra Maria Staszak, Wieslawa Jarmuszkiewicz, Ewelina Ratajczak

**Affiliations:** 1 Department of Biotechnology, Institute of Molecular and Biotechnology, Adam Mickiewicz University, Collegium Biologicum, Uniwersytetu Poznańskiego 6, 61-614 Poznań, Poland; 2 Department of Biochemistry, Institute of Molecular Biology and Biotechnology, Adam Mickiewicz University, Collegium Biologicum, Uniwersytetu Poznańskiego 6, 61-614 Poznań, Poland; 3 Department of Trace Analysis, Faculty of Chemistry, Adam Mickiewicz University, Uniwersytetu Poznańskiego 8, 61-614 Poznań, Poland; 4 Plant Physiology Department, Faculty of Biology, University of Bialystok, Ciółkowskiego 1J, 15-245 Bialystok, Poland; 5 Laboratory of Mitochondrium Biochemistry, Department of Bioenergetics, Institute of Molecular and Biotechnology, Adam Mickiewicz University, Collegium Biologicum, Uniwersytetu Poznańskiego 6, 61-614 Poznań, Poland; 6 Institute of Dendrology, Polish Academy of Sciences, Parkowa 35, 62-035 Kórnik, Poland

**Keywords:** Physiology, Plant sciences

## Abstract

Plant metal hyperaccumulators, to which *Brassica juncea* belongs, must have very efficient defence mechanisms that enable growth and development in an environment polluted with various heavy metals. *B. juncea* (Indiana mustard) v. Małopolska was exposed to the activity of trace elements such as cadmium (Cd), copper (Cu), lead (Pb), and zinc (Zn) in combinations: CuPb, CuCd, CuZn, PbCd, PbZn, and ZnCd in a concentration of 25 μM each for 96 h during control cultivation. We observed a clear tendency for metal uptake and accumulation in above-ground parts which is characteristic of hyperaccumulators. The combinations of CuCd, CuZn, and PbCd inhibited the development of the seedlings the most. The used metal combinations increased the levels of reactive oxygen species (ROS) such as: hydrogen peroxide (H_2_O_2_), superoxide anion (O_2_^.−^) and oxidized proteins in *B. juncea* organs, generating oxidative stress conditions in the cells. We determined the level of transcription of the respective defence proteins of the detoxification and antioxidant systems. We have shown that in the first 24 h of stress condiction, activation of glutamylcysteine-γ synthetase (yECS) and glutathione reductase (GR1) enzymes related to the detoxification of heavy metals is important for *B. juncea* plants. In addition, the data provide important information on how plants respond to the presence of heavy metals in the first days of stress conditions.

## Introduction

Anthropogenic activity increases year by year, including rapid industrialisation, urbanisation, the development of mining, metallurgy, fuel combustion, the use of artificial fertilizers, sewage sludge, and plastics, all of which accelerates the release of various toxic trace metals into the environment. Heavy metals affect the development and growth of the whole plant because they influence many basic physiological processes, including photosynthesis and respiration^1,2^. The constant accumulation of heavy metals in the soil can pose a serious threat to living organisms, both plants and animals^3,4^. Because biological organisms are unable to degrade metals, they remain in parts of the body and in the environment^5,6^. Heavy metals are categorized as essential and non–essential. Essential metals, including copper (Cu) and zinc (Zn), have important regulatory roles in a number of biological processes, while non-essential metals, such as cadmium (Cd) and lead (Pb), possess unknown biological functions, which are potentially highly toxic to plants^5,7,8^. The toxicity level of non-essential metals depends on their concentration and their bioavailability. Bioavailability of metals depends on abiotic variables, including physical factors, for example, soil structure. It also depends on biotic elements, for example, microbial and plant species^9,10^. Heavy metals necessary in high concentrations may also be harmful to organisms^1^. To date, more than 400 plant species from different families, such as *Asteraceae, Brassicaceae, Violaceae, Poaceae,* and *Fabaceae*, have been identified as capable of tolerating high levels of heavy metals in the soil. Among plants of the *Brassicaceae* family there are almost 80 plant species with the potential for heavy metal accumulation. Many authors have confirmed that *Brassica juncea* is able to accumulate significant amounts of some trace elements, such as Pb^11^, Pb, Cd^11,12^, Se^13^, Zn, Cr, Cu, and Au^14^, Cr, Cu, Ni, Pb, and Zn^5,15^, although their translocation capacity is not as high as demonstrated for other known hyperaccumulators. *B. juncea* is a tolerant plant to heavy metals, grows fast and produces a large amount of above-ground biomass. Due to these characteristics, this species has been the target of several studies to evaluate its phytoremediation potential^16^. In most plants, metals are predominantly accumulated in the roots, but in hyperaccumulators metal concentrations are substantially higher in the aboveground parts^1^. The chelation of heavy metals, especially by phytochelatins, seems to be one of the most important mechanisms for the tolerance of *Brassica* species. In these plants the role of heavy metal transporters is much clearer and they seem to be of a fundamental importance in the tolerance to various heavy metals^16^. In the presence of metals, there is an increase in ROS generation in plant cells and oxidative damage^5^. Therefore it seems that metal hyperaccumulating plants should have extremely efficient antioxidative and detoxicative defence mechanisms, enabling growth and development in a polluted environment. Due to advances in research on levels of transcripts and metabolites involved in abiotic stress such as heavy metals, it will be possible in the future to establish a clear picture of the tolerance and defence mechanisms used in *Brassica* species^16^. Plants exposed to heavy metal stress respond by altering their cellular mechanisms and gene expression^7^. Owing to efficient detoxification mechanisms such as glutamylcysteine-γ synthetase (γECS) and glutathione reductase (GR1), plants avoid metal toxicity, but suffer from oxidative stress with increasing stress levels. This is manifested by excessive production of reactive oxygen species (ROS), and increases in the degree of lipid peroxidation and the level of oxidized proteins. Plant cells are equipped with an antioxidant defence system to minimize oxidative stress, such as the antioxidant enzymes: superoxide dismutase (SOD), catalase (CAT) and ascorbate peroxidase (APOX). Most of the available literature data present the results of impact studies on individual trace elements per plant, whereas they often grow in soil containing various metal ions. Therefore, in our study, we used a two-element combinations of metals to assess their net effect on the seedlings of *B. juncea*.

The main objectives of this study were to determine how stress conditions caused by contamination with bimetals in *B. juncea* organs influence (i) the increase in the biomass of mustard seeds, (ii) the accumulation of trace metals and micronutrients, (iii) translocation of metals from roots to above-ground parts, (iv) the level of oxidative stress, (v) the level of transcription of enzymes of the detoxificative and antioxidative system in the first 24 h of exposure to abiotic stress.

## Results

### Levels of metal accumulation

**Figure 1:**
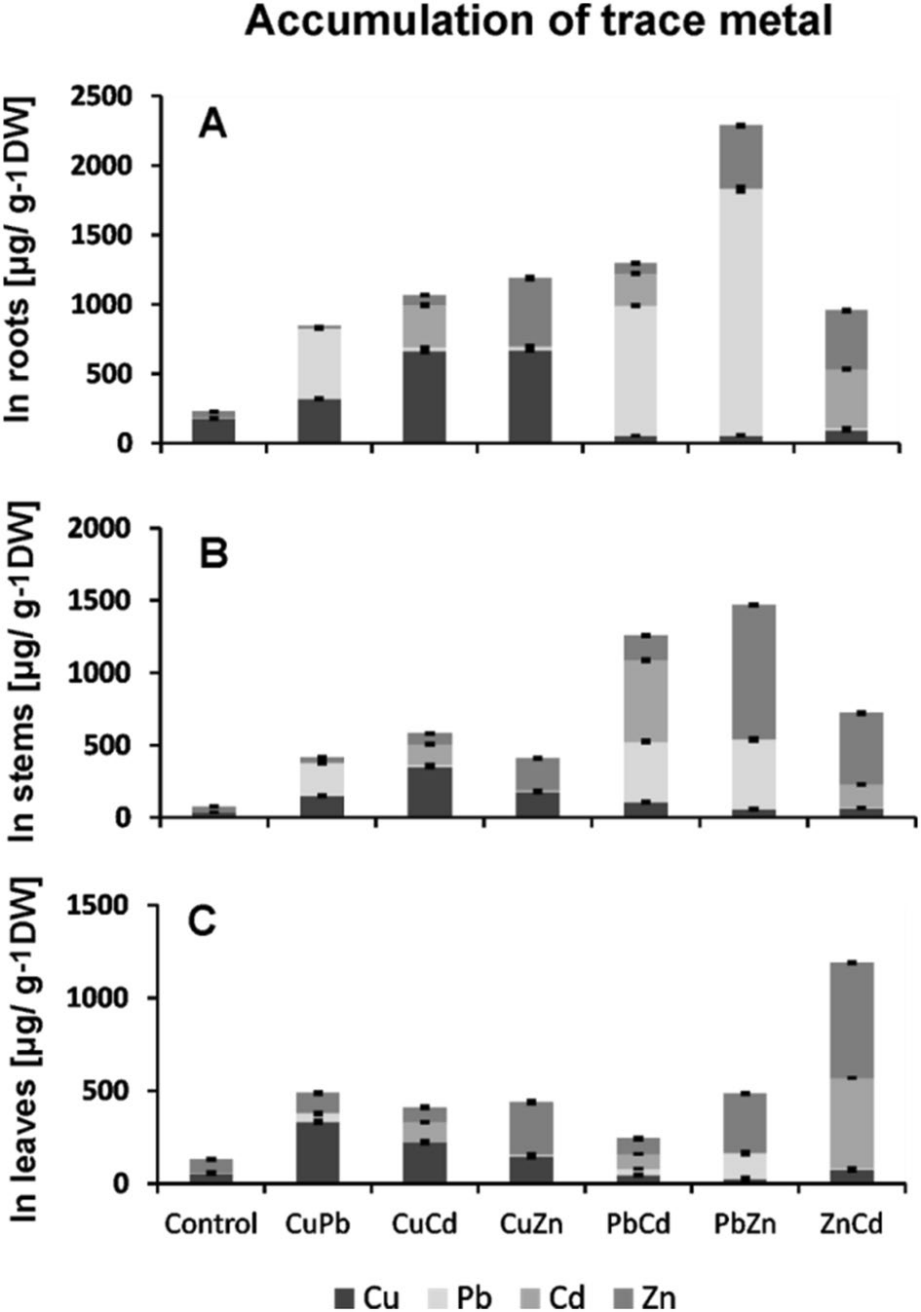
Accumulation of metals: Pb, Cu, Cd, and Zn in the roots (**A**), stems (**B**), and leaves (**C**) of *B. juncea* v. Malopolska seedlings. Plants were grown in Hoagland’s medium and treated metal bicombinations: CuPb, CuCd, CuZn, PbCd, PbZn, and ZnCd. Metal solutions Pb(NO_3_)_2_, CuSO_4_, CdCl_2_, and ZnSO_4_ were applied at a 25 μM concentration of each. Mean values of three replicates (± SD).

The levels of metal accumulation in the roots, stems, and leaves of B*. juncea* were determined using the inductively coupled plasma mass spectrometry (ICP-MS), (Fig. 1). The highest accumulation of Pb was observed in the roots, especially in the case of the PbZn combination and it reached over 70% of all metals taken, while to the above-ground parts, Pb was transported in the amount—20% in stems, 6% in leaves. Cd ions were transported to the above-ground parts in plants treated with ZnCd the most: roots 39%, 15% shoots, and 46% leaves. An equally high accumulation of Pb and Cd metals was observed in the shoots of plants treated with PbCd, amounting to 45% for Cd and 39% for Pb.

### Effect of the metal uptake on micronutrient accumulation in plants *B. juncea*

**Figure 2:**
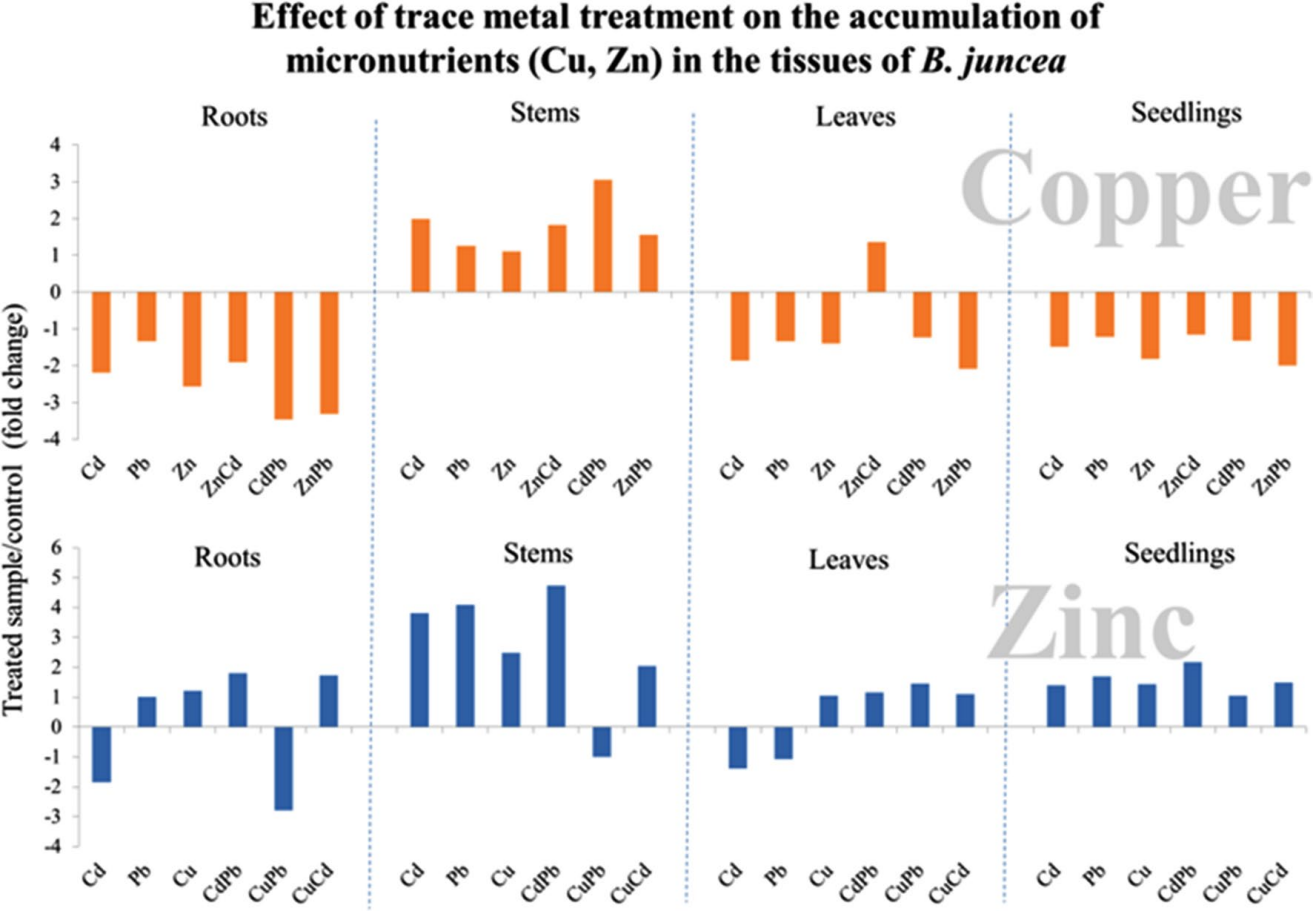
Accumulation of essential microelements: Cu, Zn in *B. juncea* v. Malopolska organs (roots, stems and leaves). Plants were grown in Hoagland’s medium and treated with bimetal combinations: CuPb, CuCd, CuZn, PbCd, PbZn, and ZnCd. Metal solutions Pb(NO_3_)_2_, CuSO_4_, CdCl_2_, and ZnSO_4_ were applied at a 25 μM concentration of each. Mean values of three replicates (± SD).

**Figure 3:**
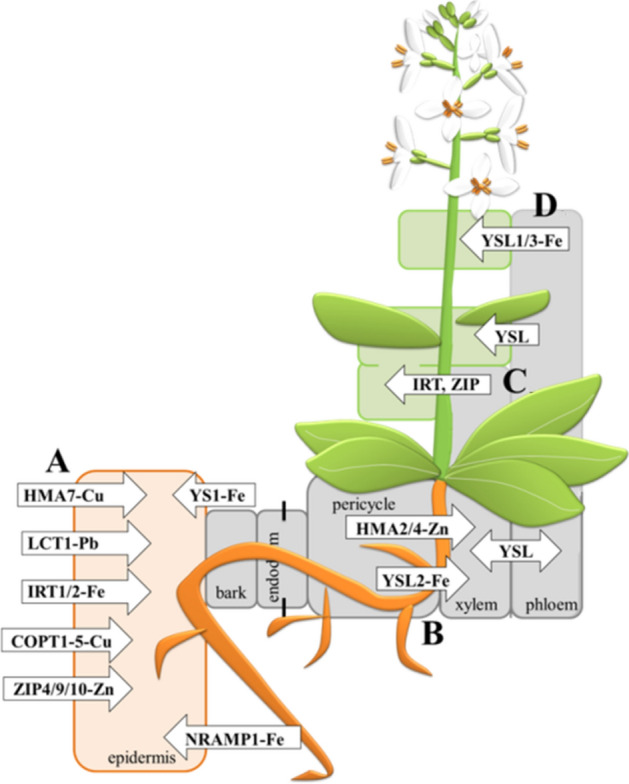
Generic diagram of the localisation of postmembrane trace metal transporters in higher plants. Part (**A**) uptake of metals to the roots, (**B**) transport to xylem/phloem, (**C**) transport to the leaves, (**D**) transport to the seeds (metals transporters: HMA2/4 for Zn; ITR1/2 for Fe; HMA5 for Cu, ZIP family, LCT1 channels, Nramo family for Fe, Ca and Zn; COPT1 for Cu, YSL family for Fe).

Our research on the effect of treating mustard seedlings with trace metals on the plant's ability to uptake micronutrients yielded results (Fig. 2). We expected that the presence of high concentrations of exogenous metals in the medium would lower the content of copper and zinc. Meanwhile, during the treatment with metals, zinc uptake increases and copper decreases in whole plants, and significant differences are observed at the level of individual organs. Under the tested conditions, there is a clear tendency for microelements to accumulate in the stems, both for Cu and Zn. This may be partly explained by an increase in apoplastic transport to the conducting bundles and the opening of nonspecific ion channels in the presence of trace element ions competing for specific transporters. The metal accumulation profile is similar in roots and leaves, where the metal uptake into cells from the solutions is observed—soil in the roots and apoplastic transport in leaves. Transport to the conductive bundles requires different transporters (Fig. 3).

### Biomass and morphological changes

**Figure 4:**
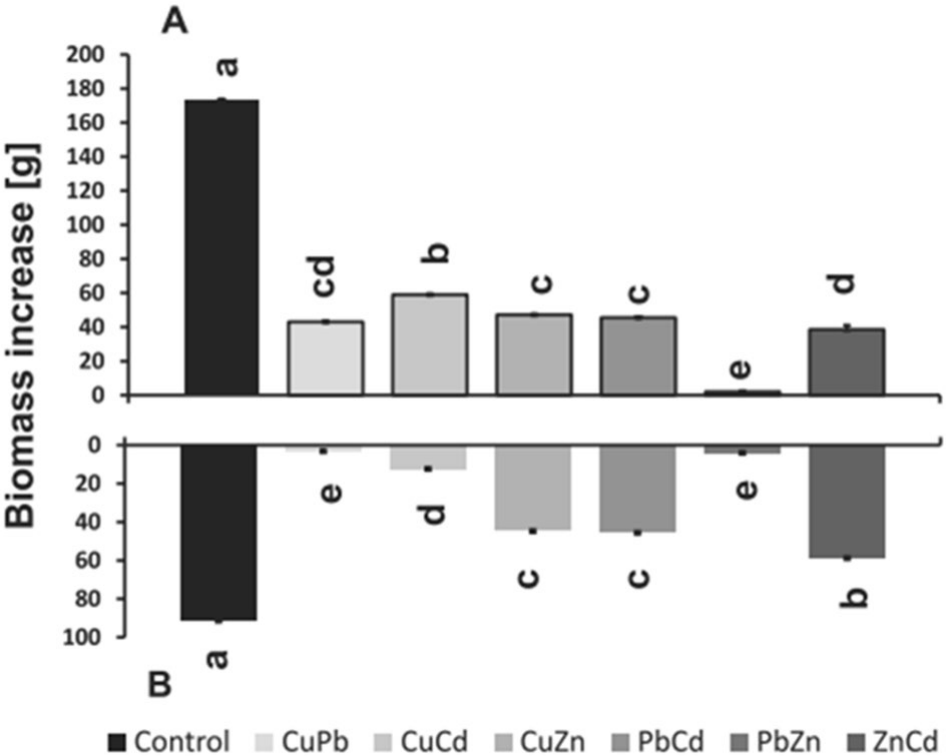
Changes in biomass of *B. juncea* roots and seedlings (**A**) and roots (**B**) treated with trace metals: CuPb, CuCd, CuZn, PbCd, PbZn, and ZnCd after 96 h of cultivation. The results are expressed as the mean ± standard deviation (n = 3), with results of Duncan's Test, made separately for A and B. Metal solutions Pb(NO_3_)_2_, CuSO_4_, CdCl_2_, and ZnSO_4_ were applied at 25 μM concentration of each.

The highest inhibition of seedling biomass growth was observed for *B. juncea* plants treated with combinations of CuCd, CuZn, and PbCd (Fig. 4). After 96 h of cultivation, the biomass of seedlings was about 43–59% lower than at the beginning of cultivation and 68% -76% lighter than in the control plants. The weakest inhibitory effect was observed for the ZnCd-treated seedlings, because after 96 h of treatment, the seedlings were 38% heavier than at the beginning of the cultivation and only 20% lighter than the control plants. We observed morphological changes in the area of the leaf blade, which were manifested by the presence of necrotic spots on the leaves and the inhibition of the growth of the leaf surface in relation to the control seedlings. Moreover, in the case of seedlings treated with CuZn and CuCd, the leaves were slightly twisted, and with CuPb, PbCd, and PbZn, there was chlorosis. The ZnCd combination caused the smallest morphological changes.

**Figure 5:**
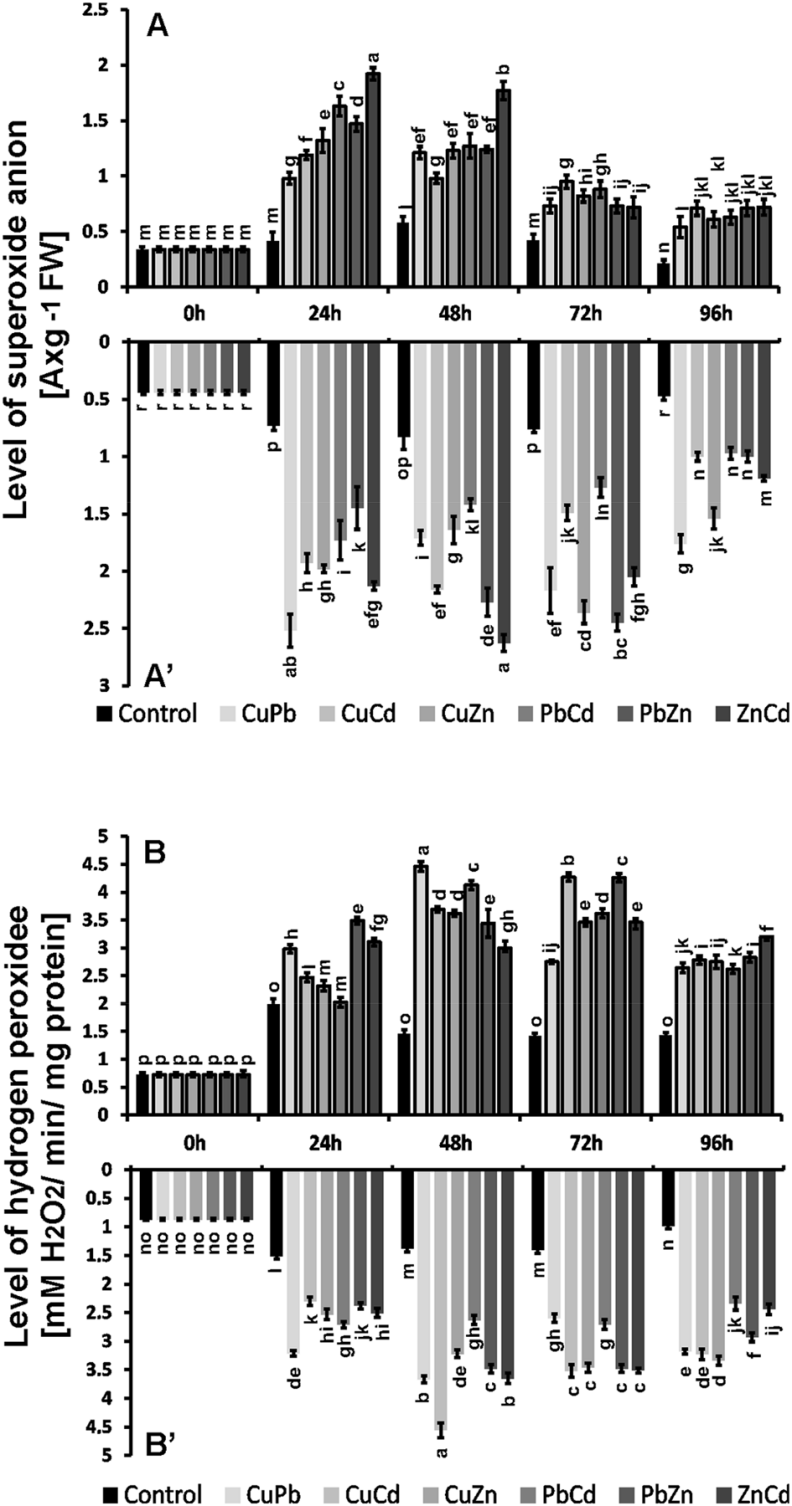
Superoxide anion (A580 g^−1^ FW; (**A**)-above-ground part, (**A'**) -root) and hydrogen peroxide (nMol H_2_O_2_ × min^−1^ × mg protein^−1^; (**B**) —above-ground parts, **B'**—root) levels in in *juncea* v. Malopolska seedlings grown in Hoagland’s medium and treated with ions in combinations: CuPb, CuCd, CuZn, PbCd, PbZn, and ZnCd after 96 h of cultivation. Metal solutions Pb(NO_3_)_2_, CuSO_4_, CdCl_2_, and ZnSO_4_ were applied at a 25 μM concentration of each. Mean values of three replicates (± SD), with results of Duncan's Test, made separately for (**A**) and (**B**).

Higher levels of O_2_^.−^ were observed in roots exposed to constant bimetal stress than in the above-ground parts, reaching maximum values between the 24 and 72 h of cultivation (Fig. 5). We observed the highest values of O_2_^.−^ in seedlings treated with the ZnCd combination, both in the roots and in the above-ground parts. A high level of O_2_^.−^ in mustard roots was also observed for the combination of CuPb (after 24 h) and PbZn (after 72 h). In the above-ground parts, the highest level of O_2_^.−^ was observed after 24 h especially for ZnCd, and then in the following days of cultivation it decreased by about 50%.

The profile of changes and the level of H_2_O_2_ values were similar in the roots and in the above-ground parts (Fig. 5). High values of H_2_O_2_ were obtained after 24 h of cultivation in the roots of plants treated with mixtures, especially CuCd, ZnCd, and CuPb, and they were 2–3 times higher than in control plants. In the above-ground parts, the highest level of H_2_O_2_ was observed for the samples treated with CuPb after 48 h and the combinations of CuCd and PbZn after 72 h and they were 3–4 times higher than in the control plants. In the roots, after 96 h, a decrease in the level of H_2_O_2_ could be seen, in the above-ground parts after 72 h; however, it remained at a level 2–3 times higher than in control plants until the end of the cultivation in plants exposed to metal combinations.

**Figure 6:**
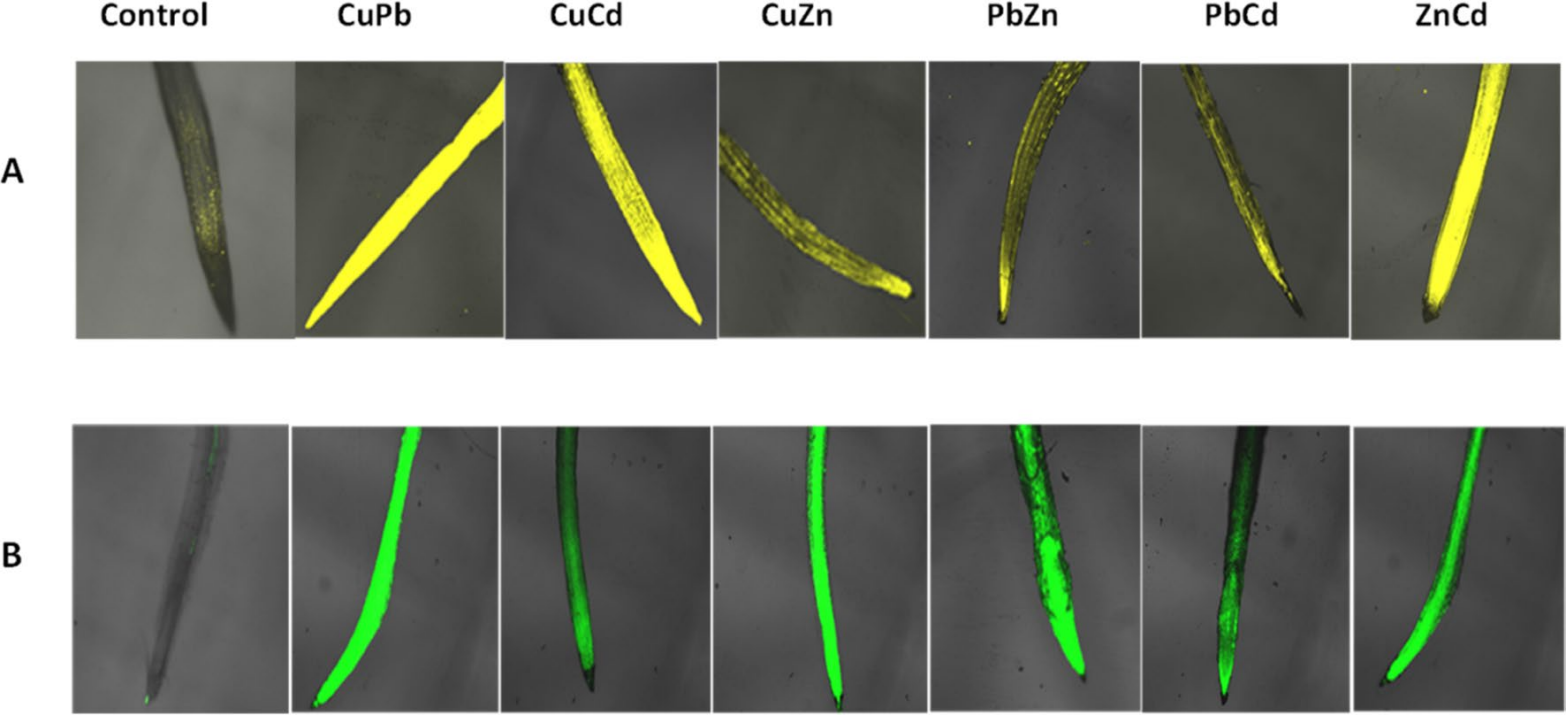
Generating of  O_2_^.^^–^ and H_2_O_2_ in *B. juncea* v. Malopolska roots using fluorescent microscopy. Plants were grown in Hoagland’s medium in the presence bimetals of 25 μmol of Pb(NO_3_)_2_, CuSO_4_, CdCl_2_, and ZnSO_4_ in combinations: CuPb, CuCd, CuZn, PbCd, PbZn, and ZnCd for 24 h. The roots of treated plants and control stained with DHE for 12 h (**A**) and DCFH-DA for 4 h (**B**).

The profile of changes in O_2_^.−^ and H_2_O_2_ levels observed with the confocal microscope was consistent with the results obtained spectrophotometrically. The most intense DHE fluorescence, indicating the presence of O_2_^.−^, was observed in the roots of *B. juncea* treated with CuPb, CuCd, and ZnCd, while the highest generation of H_2_O_2_ was observed in the roots exposed to CuPb, CuZn, PbZn, and ZnCd after 24 h of cultivation (Fig. 6).

### Level of protein oxidation

**Figure 7:**
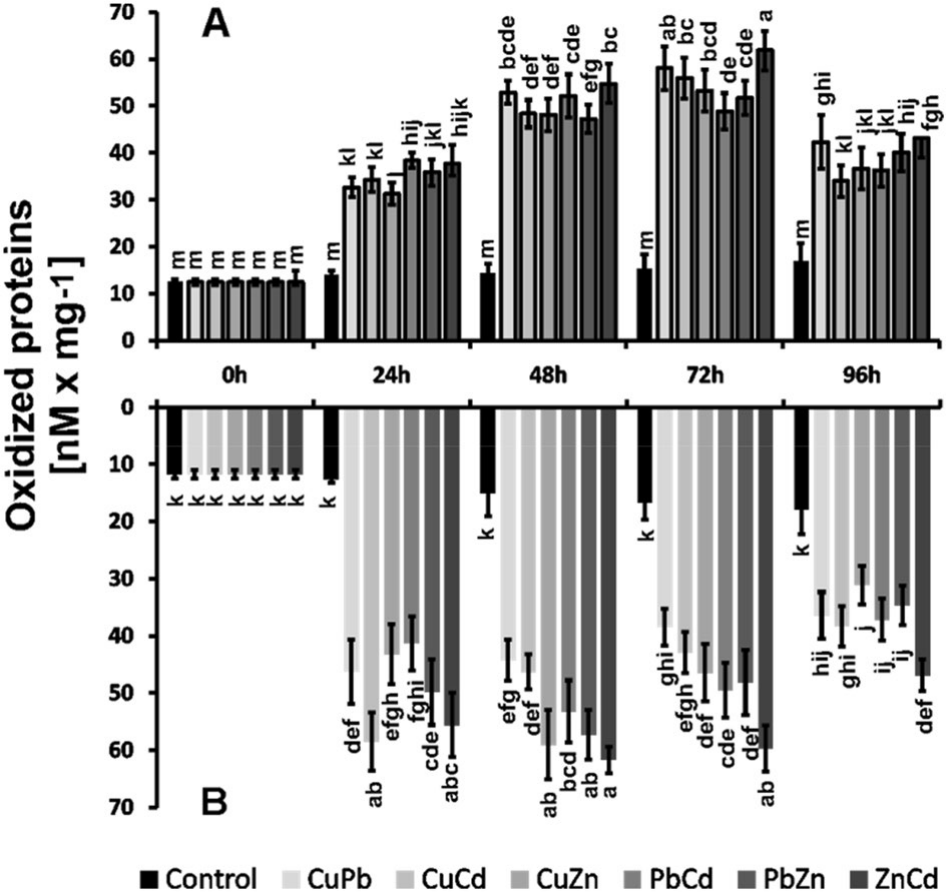
Oxidized protein level above-ground parts (**A**) and roots (**B**) of *B. juncea* v. Malopolska seedlings grown in Hoagland’s medium and treated with ions in combinations: CuPb, CuCd, CuZn, PbCd, PbZn, and ZnCd after 96 h of cultivation. Metal solutions Pb(NO_3)2_, CuSO_4_, CdCl_2_, and ZnSO_4_ were applied at a 25 μM concentration of each. Mean values of three replicates (± SD), with results of Duncan's Test, made separately for (**A**) and (**B**).

The levels of protein oxidative modification imposed by the bimetal treatment were 2–4 times higher in the roots and the above-ground parts compared to the control plants (Fig. 7). In the roots, the level of oxidized proteins reached its maximum after 48 h, while in the above-ground parts after 72 h of exposure to combination bimetals.

### Levels of gene transcripts

**Figure 8:**
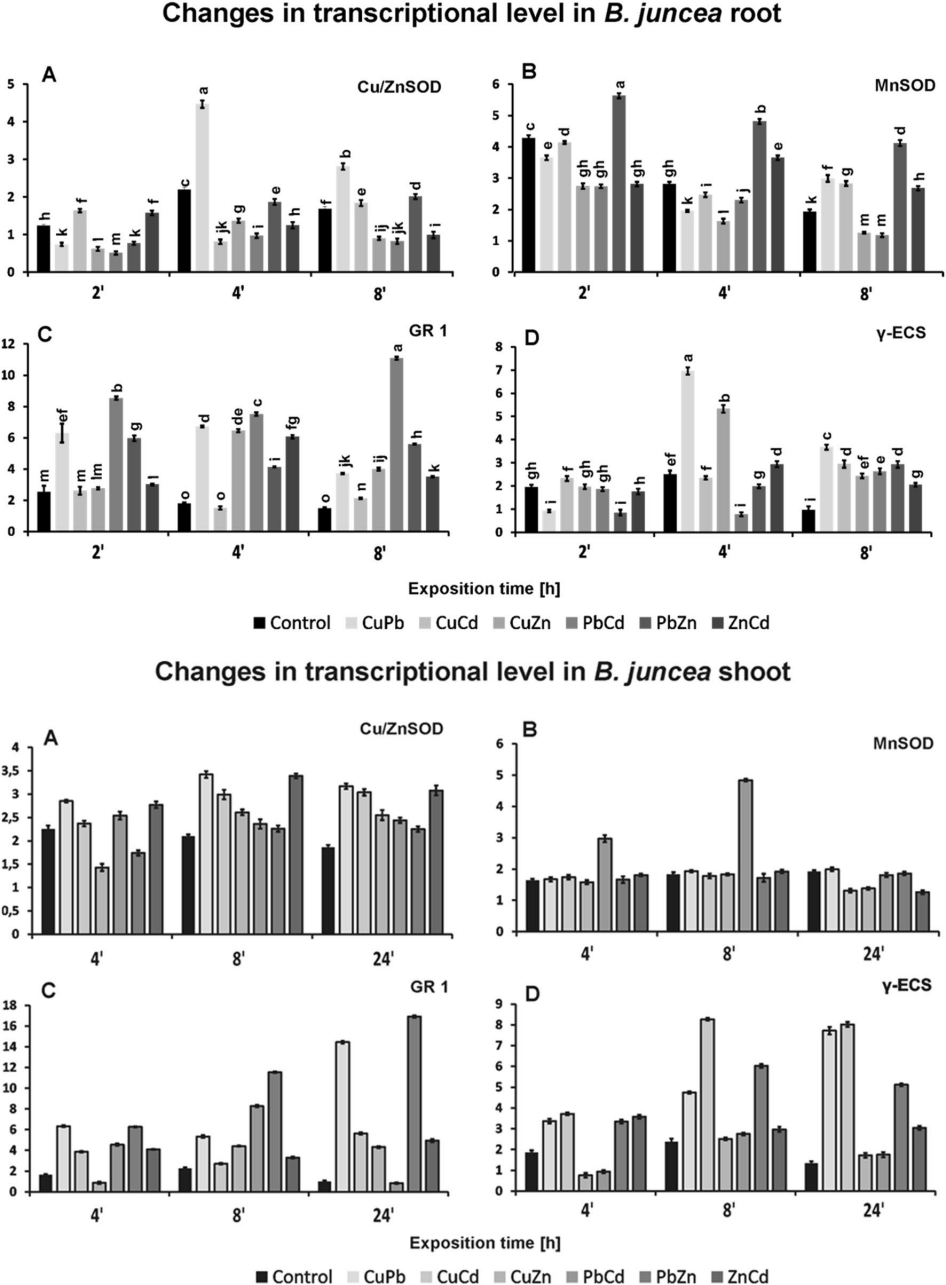
Transcriptional levels of genes encoding antioxidative (CuZnSOS—**A**, MnSOD—**B**) and detoxitative (GR1—**C**, γECS—**D**) enzymes in roots and shoots of *B. juncea* v. Malopolska seedlings. Plants were grown in Hoagland’s medium and treated with bimetals combinations: CuPb, CuCd, CuZn, PbZn, PbCd, and ZnCd. Metal solutions Pb(NO_3_)_2_, CuSO_4_, CdCl_2_, and ZnSO_4_ were applied of each at a 25 μMol concentration. Enzymes chosen for the experiment were amplified using semi-quantitative RT-PCR. Primers were designed for *Arabidopsis thaliana* genes: CSD1 for CuZnSOD, MSD1 for MnSOD, GR1 for GR, and γECS.

To estimate the changes at the level of transcripts of genes encoding CuZnSOD, MnSOD, yECS, and GR1, we used the electrophoretic separation technique and the CpAtlas program (Fig. 8).

An almost double increase in the expression of the gene encoding CuZnSOD was observed in the roots after 4 and 8 h for plants treated with the CuPb combination, while in the above-ground parts, mainly for combinations with Cu, i.e. CuPb, CuZn and CuCd, there was an increase of about 50% after 4 h. In the early hours of the stress factors activity, that is, after 2 and 4 h, no significant changes in the transcription levels of the gene encoding MnSOD in the roots were observed as compared to the control plants. Only after 8 h, an induction of the MnSOD gene expression in the roots by the ZnPb combination was observed. On the other hand, in the above-ground parts, a significant increase in the level of transcription was noted, over 2 times, for the combination of CdPb after 4 and 8 h of cultivation.

By analyzing the changes in the expression of the GR1 gene, a clear induction of gene expression in the roots was observed in plants treated with mixtures containing Pb, i.e. CuPb, CdPb, and ZnPb at all tested times (2–5 times), in the above-ground parts with CuPb and ZnPb (2–10 times).

When analyzing the changes in the expression of the gene encoding the γECS a 2–3  times, an increase in the level of transcription was observed in roots after 4 h and 8 h of exposure to combinations of CuPb and CuZn.

In the above-ground parts, a clear gene induction was observed, expressed by more than a twofold increase in the transcript level after 8 h for the samples treated with combinations of CuCd, CuPb, ZnPb, ZnCd. Plants treated with CuCd, CuPb, ZnPb still showed a very high level of transcription, three–fivefold also after 24 h.

## Discussion

Abiotic stresses, including the presence of trace metals in soil, are estimated to be the main cause of global crop yield reduction by ca. 70% and thus are considered a great constraint to crop production^5^. *B. juncea* is the oldest cultivated crop for eating, oil food, and biodiesel production^17^. Therefore, it is very important to understand its defence mechanisms against the toxic effects of trace metals. Plants, especially hyperaccumulating plants, have developed very efficient defence mechanisms, both in terms of metal detoxification and removing damage resulting from the presence of metals, including oxidative damage^18^. Thus, it is important to learn about these mechanisms and determine which one whether detoxifying or antioxidant, is activated first at the level of transcription of genes encoding suitable enzymes. Understanding these mechanisms will make it possible to determine the role of both the detoxification and antioxidant systems under the conditions of abiotic factors. In our study, we found that in the case of *B. juncea* v. Malopolska, all the mentioned combinations of metals used in the concentrations of 25 μM each displayed moderate phytotoxic properties. The greatest reduction in biomass growth was caused by the mixtures of CuCd, CuZn and PbCd, only the ZnCd combination caused no its significant reduction. At the same time, we observed an intense uptake and accumulation of Cd in the above-ground parts of plants treated with ZnCd. These results demonstrate a synergistic interaction of these metals in *B. juncea* during translocation in shoots. Other researchers have also noted that *B. juncea* accumulates metals such as: Cd, As, Pb and Cu in the aboveground parts^15^, whereas different authors^19^ reported that Zn induced a decrease in the Cd uptake and a simultaneous increase in the Zn accumulation in tomato plants. This suggested strong competition between Zn and Cd for the same membrane transporters. Using the ICP-MS method, we recorded higher translocation and accumulation of Pb, Cd, and Zn in the above-ground parts of *Brassica juncea* than in roots. Other authors^20^ have also noted levels of heavy metals in *B. juncea* shoots and the order of bioaccumulation was as follows Cu > Cd > Pb > Cr. Similarly, Rajput et al.^21^ found, that the ability to hyperaccumulate different pollutants can be correlated either positively (Cd–Zn, Pb–Zn, Co–Cu, Cd–Pb) or negatively (Cu–PAHs, Co–Cd, Co–PAHs, Ni–PAHs, Cu–Ni, Mn–PAHs). The authors^21^ suggest that the metals Cd, Pb and Zn are very similar to each other in their soil biochemistry and migration patterns, and hence they can use the same transporters. It was found that Cd, Pb, and Zn are carried into vacuoles by the same P-type ATPase HMA3, suggesting that these metals are hyperaccumulated by the same mechanisms^21^. Trace metals can compete with each other for metal transporters, e.g. Cd^2+^ ions have physicochemical properties similar to essential divalent metal ions and use their transporters (HMA2/4, ZIP4, etc.). This is an example of direct competition between metals for pathways to enter cells. Lead ions are transported mainly apoplastically or through channels for bivalent ions. Calcium ions enter the plant in a similar way; therefore, the increased concentration of lead ions may destabilize the calcium metabolism^1,22,23^. We observed an increase in zinc uptake and a decrease in copper in individual organs. The inhibition of biomass and plant growth by trace metals may be caused by many factors, such as water stress, which leads to a reduction in stomatal conductivity, lower transpiration and relative water content in leaves^5,7^. Plants, through the root cortex tissues, take metals from the soil solution due to their similarity with the same essential micronutrients such as Zn or Cu, and then symplastically or apoplastically they pass to the xylem and are transported to the shoots and stored in vacuoles^7,20^. Milner and Kochian^24^ found that Zn was bound to histidine and organic acids inside the roots and in the free ionic form of Zn^2+^ in xylem juice. In contrast, the Zn in shoots was found mainly in combination with organic acids, and only some parts of it were in the free ionic form. This suggests that the metal ions are transported from the roots in the chelated form as free ions and then stored again in the shoots, where they are bound by the chelates^9^. Other authors have also noted that Zn and, to a lesser extent, Cd are mobile and can be transferred by phloem to the above-ground parts of plants and accumulate in meristems (root tips, shoot tips, axillary buds)^23^. Israr et al.^25^ concluded that the co-presence of metals (Pb + Cu, Pb + Ni, Pb + Zn, Cu + Ni, Cu + Zn, Zn + Ni, Pb + Ni + Cu + Zn) results in a greater reduction in plant biomass than exposure to single metals because of the occurrence of a synergistic or additive response. We observed the accumulation of the metals Cd, Zn, and Pb in the above-ground parts of plants in an amount 50% greater than in the roots. These results indicate an increase in apoplastic transport to the conducting bundles and the opening of nonspecific ion channels in the presence of trace element ions competing for specific transporters. There was a synergistic response between metals in the combinations PbZn and ZnCd, resulting in an increased accumulation of the two metals in the above-ground parts. *B. juncea* is a good accumulator of trace metals, especially Cd and could be used for the remediation of Cd from contaminated soils, because of its ability to translocate large amounts of this metal into its stems and leaves^15^. Trace metals can cause plant growth reduction by decreasing photosynthetic rates, as they disturb mineral nutrition and water balance, change hormonal status, and affect the membrane structure and permeability and chlorophyll content^26,27^. We noted that the presence of other metals in the medium changes the mineral balance of the whole *B. juncea* plant. Heavy metals may cause severe toxicity in plants by disturbing essential groups such as -SH of enzymes leading to their inactivation, destroying the integrity of important biomolecules, modifying some macromolecules, replacing essential metal ions from structural formulae of biomolecules, and enhancing the generation of ROS^7,25^.

We observed a significant increase in ROS levels compared to the control plants in all plants treated with the combination of heavy metals, both in the roots and in the aerial parts. The highest values ​​were observed in the organs of plants treated with ZnCd, CuPb, CuCd and PbZn. Therefore, it can be concluded that the most pro-oxidative are the combinations containing the metals Cu and Zn.

Toxic metals/metalloids are categorized into redox-active (Fe, Cu, Cr, etc.) and redox inactive (Cd, Pb, Zn, Ni, Al, etc.). Metals with higher redox potential than biological molecules (such as Fe^2+^ and Cu^2+^) participate in biological redox reactions and ultimately induced the ROS directly through Haber–Weiss and Fenton reactions. Other metals/metalloids such as Cd, Pb, Al, Ni, Zn, etc. cause the ROS generation by impairing the antioxidant defence system (mainly glutathione pool), inducing NADPH oxidase, displacing obligatory cations from binding sites of enzymes and activating the Ca-dependent system^28,29^. Other authors have also reported higher H_2_O_2_ production in *B. juncea*: under the influence of Cd it was twofold higher after 7 days^18^, for Cr, Si and Cr + Si it was about 1.5-fold higher^30^, for Cd about tenfold after 5 days^31^ and for As about 2-fold higher^32^. Also, an over threefold increase in O_2_^.−^ was observed in 10-day-old *B. juncea* seedlings treated with Pb, and with Cd over twofold^33^. Similarly, a two-fold increase in O_2_^.−^ and H_2_O_2_ was observed in *B. juncea* seedlings treated with Cd at a concentration of 100 mg/kg for 45 and 60 days^34^. The fluorescence signal indicating the presence of O_2_^.−^ and H_2_O_2_ was observed mainly in the meristematic and elongation zones of *B. juncea* roots, which was also confirmed by other authors in *B. juncea* roots exposed to 50 μM Cu^35^. This is probably due to the fact that these zones are most exposed to the toxic effects of metals. An increasing histochemical detection of O_2_^.−^ and H_2_O_2_ was observed in the leaves of *B. juncea* seedlings treated with Cd^36^.

The increase in ROS in the roots and above-ground parts of *B. juncea* resulted in an oxidative stress condition. Under these conditions, pro-oxidative-antioxidant reactions towards oxidation are disturbed^37^. Consequently, this led to a two–fourfold increase in the level of oxidized proteins in *B. juncea* organs. ROS plays a key role as a signaling molecule in plants and controls the physiological functions of plants under stress. Therefore, the balance between ROS production and their elimination is of great importance for the proper functioning of plant cells^28^.

The rapid increase in oxidative stress conditions in cells requires the rapid activation of the antioxidant and the detoxification system, both at the level of activity and the expression of key enzyme genes. In our study, we observed differences in the induction of gene expression encoding enzymes depending on exposure time and metal combination. On the basis of our research, induction of gene expression was observed: CuZnSOD mainly by combinations with copper, MnSOD mainly by combinations of PbZn, and GR1, and yECS mainly by combination containing Cu and Pb ions. Other authors have also^38^ observed an increase in the transcript amounts of γ-glutamylcysteine synthetase (γ-ECS), an enzyme which catalyses the first step of the glutathione (GSH) synthesis, in *B. juncea* plants under Cu stress. For the genes encoding CuZnSOD, GR1 and y-ECS, there was a greater increase in expression in shoots than in roots. This is probably due to the fact that hyperaccumulators are characterized by a higher accumulation of metals in the above-ground parts of the plants. Plants respond to abiotic factors such as heavy metal stress via immediate change in expression of the stress responsive genes at the transcriptional level^39^. The superoxide radical dismutation reaction is one of the first stages of the cascade of processes leading to the detoxification of ROS^5,37^. Therefore, the induction of genes encoding SOD, including CuZnSOD and MnSOD, is a very important factor determining the acclimatization of plants to stressful conditions^5,40^. However, it seems that *B. juncea* plants, which are hyperaccumulators, tolerate oxidative stress conditions well and do not show the need to induce the antioxidant system at the level of protein transcription. Our results indicate that during the first 24 h of exposure to metal stress, gene expression is induced, mainly the detoxification enzymes GR1 and yECS. Naturally, under metal stress, ROS are generated—hence the need to induce the expression of genes encoding antioxidant proteins. In our previous research, we observed an increase in the level of the gene encoding CuZnSOD in *B. juncea* plants treated with single copper, zinc, and lead ions the highest after 4 h in the roots and after 8 h in the above-ground parts, and MnSOD in the roots after 8 h exposure to Zn and Pb ions^5^. Other authors^41^ using microarray results found that the Cd stress response in *Brassica nigra* plants after 72 h was much stronger in roots (88 genes showing increased or decreased mRNA levels) than in shoots (24 responding genes). They detected an increase in the expression of genes encoding y-ECS and PCS (phytochelatin synthase) in the roots and leaves of the tolerant and nontolerant ecotypes of *B. nigra*. CuZn superoxide dismutase (CSD1), catalase (CAT3 and CAT1), and peroxidase genes were downregulated, indicating regulation of hydrogen peroxide contents. Also, a significant effect of metal Cd stress on the induction of gene expression related to GSH (glutathione) metabolism in *B. juncea* using Comparative Transcriptome Profiling was noted by Thakur et al.^42^. These authors observed that among the transcripts mapping to GSH metabolism, the gene expression of a total of ten important components including glutathione S-transferase (GST), glutathione synthase (GSS), and gamma-glutamyltranspeptidase (GGT) was found to be modulated by cadmium. A total of 66 different transcripts encoding GST were found to be significantly upregulated. Additionally, other authors^43^ observed in *Brassica chinensis* L. cultivar Aikangqing (a Cd-tolerant cultivar) an increase in expression of genes encoding y-ECS and GR in shoots after exposure to Cd ions. The authors postulate that in response to Cd stress in plants, there is overexpression of enzymes related to sulphur assimilation, thus increasing the tolerance to heavy metals. The addition of S in the experiment identified an increase in expression of defence enzyme genes. S uptake results in the initial formation of the first solid product, cysteine, which is needed for the synthesis of GSH, a low molecular weight antioxidant. GSH, in turn, is involved in both the ASA-GSH cycle and the biosynthesis of phytochelatins to regulate heavy metal detoxification. Therefore, whether the regulation of S assimilation related to heavy metal toxicities is essential in plant defence mechanisms still requires research^42,43^. Undoubtedly, however, exposure to toxic metal ions or high concentrations of non-toxic ions triggers stress reactions in plants that require adaptation at various levels: structural, physiological, biochemical and molecular. Based on our present results, it seems that triggering defence mechanisms related to detoxification of heavy metals is crucial for *B. juncea* plants. However, we have previously reported^6^ that plants are not adequately protected by the detoxification system because trace metals penetrate areas with high metabolic activity, such as the cytoplasm, mitochondria, or cell membrane—hence, the strong need for the mutual cooperation of two defence systems of plants: detoxicative and antioxidative.

## Materials and methods

### Plant material

The plant material was grown according to Malecka et al.^5^. Seeds of *B. juncea* v. Malopolska were grown in Petri dishes for 7 days under optimal conditions. Next, young seedlings were cultivated hydroponically on Hoagland’s medium for one week in a growth room with a 16/8 h photoperiod, day/night at room temperature and light intensity of 82 μmol m^2^ s^−1^. After that, we changed the medium to 100 × -diluted Hoagland’s solutions. Next, on 14 day old seedlings a trace metal solution was applied in combination: CuPb, CuCd, CuZn, PbCu, PbCd, Pb Zn ,and ZnCd at a concentration of 25 μM of each. We used a solution of Pb(NO_3_)_2_, CuSO_4_, CdCl_2_, ZnSO_4_ in the cultivation. For our studies, we chose the concentration and 25 μM because according to our earlier experiments^1^, these are the concentrations at which metals, both essential and non-essential, cause negative changes in Brassica plants, but are not lethal. The roots and shoots of *B. juncea* were cut off after 0, 24, 48, 72, and 96 h of cultivation. To eliminate trace elements adsorbed at the root surface, they were dipped sequentially in cold solutions of 10 mM CaCl_2_ and 10 mM EDTA for 5 min each. Next, roots and shoots were rinsed three times with distilled water, frozen in liquid nitrogen, and stored at − 80 °C until the molecular analysis. All other experiments were performed on a freshly harvested plant material.

### Biomass increase

The changes in fresh biomass of *B. juncea* seedlings: control and treated with bimetal combinations: CuPb, CuCd, CuZn, PbCu, PbCd, Pb Zn, and ZnCd were measured using the Radwag scale after 0, 24, 28, 72 and 96 h of cultivation.

### Accumulation of trace elements

Roots, stems, and leaves of *B. juncea* were collected after 72 h of treatment with trace metals. Plant samples were rinsed with distilled water, dried at 70 ± 2 °C, and milled. Next, the dried samples were digested in close microwave digestion oven Ethos One (Milestone S.r.l., Sorisole BG, Italy). Digestion was carried out as follows: 300 mg of dry samples were accurately weighed into the microwave vessels and then 3 ml of 65% HNO_3_ and 1 mL of 30% H_2_O_2_ were added. After that, samples were transferred to 10 mL flasks filled with deionized water. Digested samples were quantitatively transferred into polypropylene tubes and diluted with demineralized water (Direct-Q 3UV, Merck, Darmstadt, Germany). The determination of trace metal accumulation was performed using SN-ICP-MS (solution nebulisation inductively coupled plasma mass spectrometry) model Elan DRC II, (PerkinElmer Sciex, Toronto, ON, Canada). More detailed information about the ICP-MS operation conditions, settings, and quality assurance is provided in Konkolewska et al.^10^.

The validity of the analytical method was assessed by analyzing the certified reference material NIST SRM 1570a Trace elements in Spinach Leaves (National Institute of Standards and Technology, Standard Reference Material, Gaithersburg, MD, USA) 10.

### Measurements of the level of reactive oxygen species

Superoxide anion content was determined according to Doke^44^ at 580 nm. The plant roots and above-ground parts (0.5 g) were placed in the test tubes and filled with 7 mL of mixture containing 50 mM phosphate buffer (pH 7.8), 0.05% NBT (nitro blue tetrazolium) and 10 mM of NaN_3_. Next, the test tubes were incubated in the dark for 5 min, and then 2 mL of the solution were taken from the tubes heated at 85 °C for 10–15 min, cooled in the ice for 5 min and the absorbance was measured spectrophotometrically (SHIMADZU UV-1800, Japan) at 580 nm against the control.

Hydrogen peroxide content was determined according to Patterson et al.^45^. The plant roots and above-ground parts were homogenized in 5% TCA (Trichloroacetic acid). The homogenate was centrifuged twice at 13,000*g* for 20 min. The level of hydrogen peroxide was determined in the supernatant by the spectrophotometric method at 508 nm. The reaction mixture contained: 50 mM phosphate buffer (pH 8.4), reagent containing 0.6 mM 4-(-2 pyridylazo) resorcinol, 0.6 mM potassium-titanium oxalate in (1:1). The corresponding concentration of H_2_O_2_ was determined against the standard curve of H_2_O_2._

### In situ detection of superoxide anion and hydrogen peroxide

The roots from plants exposed to metals Pb(NO_3_)_2_, CuSO_4_, CdCl_2_, and ZnSO_4_ in combinations: CuPb, CuCd, CuZn, PbCd, PbZn, and ZnCd for 24 h and control were cut and submerged for 12 h in 100 µM of CaCl_2_ containing 20 µM of dihydroethidium (DHE, pH 4.75; samples for superoxide anion radicals) according to Yamamoto et al.^46^ or 4 µM dichlorodihydrofluorescein diacetate (DCFH-DA) (samples for hydrogen peroxide) in 5 mM dimethyl sulfoxide (DMSO) according to Afzal et al.^47^. After rinsing with 100 µM of CaCl_2_ or 50 mM phosphate buffer (pH 7.4), the roots were observed with a confocal microscope (Zeiss LSM 510, Axiovert 200 M, Jena, Germany) equipped with no. 10 filter set (excitation 450–490 nm, emission 520 nm or more).

### Determination of the level of protein oxidation

The method of Levine et al. was used to determine the level of oxidized proteins^48^. Roots and above-ground parts (0.5 g) were incubated with isolation buffer containing 0.1 M Na phosphate buffer, 0.2% (v / v) Triton X-100, 1 mM EDTA and 1 mM PMSF. After centrifugation at 13,000 × *g* for 15 min, supernatants (200 µl) were mixed with 300 µl of 10 mM DNPH in 2 M HCl. The blank was incubated in 2 M HCl. After 1 h of incubation at room temperature, the proteins were precipitated with 10% (w/v) trichloroacetic acid (TCA) and the pellets were washed three times with 500 µl of ethanol / ethyl acetate (1: 1). The pellets were finally dissolved in 6 M guanidine hydrochloride in 20 mM potassium phosphate buffer (pH 2.3) and the absorption was measured at 370 nm. The carbonyl content was calculated from the molar absorption coefficient of the aliphatic hydrazones, 22,000 m–1 cm^−1^.

### Protein quantification

Total soluble protein contents were determined according to Bradford^49^, using the Bio-Rad assay kit with bovine serum albumin as a calibration standard.

### Isolation of total RNA and RT-PCR

Isolation of Total RNA and RT-PCR was performed using the method described by Małecka et al.^5^. Roots and above-ground parts (100 mg) of *B. juncea* plants in the presence of trace metals and under control conditions were collected for total RNA isolation. The RNA was isolated with TRIzol reagent and tested spectrophotometrically for purity at 260 and 280 nm. Then, RNA was reverse-transcribed with oligo (dT) primers using the RevertAid Reverse Transcriptase Kit (Thermo Science, Lithuania, European Union) after DNA was treated with DNase I (Thermo Science).

The primers used for qPCR are listed in Table 1.

**Table 1:** The primers used for qPCR.

GENE	PRIMER *Forward*	PRIMER *Reverse*	Gene accession number
TUB1	GTGATTGCTTGCAGGGTTTT	CAGAATACGGAAGCAAATGTCA	X54844.1
GR1	AGGGTTGGAGAATGTTGGTG	CAATAGGTGGCTGGGAGAAA	X98274.1
CSD1	GGAACTGCCACCTTCACAAT	CGTTTTCAACCACGTCCTCT	U30841.1
MSD1	CCTTGCTCCTGTCAAGGAAG	TTCAGATAATCCGGCCTCAC	M63003.1
γ-ECS:	TGTGGCTGAAGATGTCCTGA	CACACATCGAAATCGTCCAG	AF128455.1

As a reference gene, the gene encoding tubulin was used. PCRs were performed with 28 (BjGR1), 30 (BJMnSOD), and 34 (BjCuZnSOD and Bjγ-ECS) cycles of denaturation, 95 °C for 30 s; annealing primers, 53 °C for 30 s; and elongation, 72 °C for 30 s using a 1:100 diluted cDNA template and REDAllegroTaq DNA Polymerase (Novazym, Poznań, Poland). PCR products were separated by electrophoresis on a 1.3% agarose gel with ethidium bromide in TBE (445 mM Tris–HCL; 445 mM boric acid; 10 mM EDTA; pH 8.0), visualized under UV light and photographed using the Photo Print 215SD V.99 Vilber Lourmat Set. CP Atlas 2.0 were used for densitometric analysis of relative gene expression.

### Statistical analyses

All experiments were performed in three biological and technical replicates. Calculated the mean values ± SD are provided in figures. Obtained the data were analysed statistically using IBM SPSS Statistics (Version 22 for Windows). Significant differences between treatments were analysed using ANOVA, assuming p < 0.05 as the significance threshold.

### Statement

In this study we used *B. juncea* seeds from the Palikije Plant Breeding Station in Poland and our experimental research on plants was complied with institutional, national, or international guidelines.

## Conclusions

The current study demonstrates the influence of Pb, Cu, Cd, and Zn in binary combinations on the metal uptake, biomass growth, level of oxidative stress, and the level of transcriptions of detoxicative and antioxidative enzyme systems of *B. juncea*. Plants accumulated high amounts of trace metals in the above-ground parts, especially Cd using combination of ZnCd. The presence of metals resulted in a considerable reduction in *B. juncea* biomass; the highest reduction was caused by binary combinations containing Cu and PbCd. Trace metals lead to the production of O_2_^.−^ and H_2_O_2_ and increase the level of oxidized proteins. We noticed that under the conditions of metal stress, transcript level of enzymes: yECS and GR, which are associated with metal detoxification, increase, especially in shoots. No significant differences in the levels of CuZnSOD and MnSOD transcripts both in roots and in shoots were observed. The obtained results indicate that in the organs of *B. juncea* v. Malopolska, in the first hours of stress, the expression of genes encoding proteins related to the detoxicative system is induced.
